# Machine learning-based monitoring and design of managed aquifer rechargers for sustainable groundwater management: scope and challenges

**DOI:** 10.1007/s11356-024-35529-3

**Published:** 2024-11-25

**Authors:** Abdul Gaffar Sheik, Arvind Kumar, Anandan Govindan Sharanya, Seshagiri Rao Amabati, Faizal Bux, Sheena Kumari

**Affiliations:** 1 Institute for Water and Wastewater Technology, Durban University of Technology, Durban, 4001 South Africa; 2 Department of Civil Engineering, Indian Institute of Technology Bombay, Mumbai, 400076 India; 3 Department of Chemical Engineering, Indian Institute of Petroleum and Energy, Visakhapatnam – 530 003, , Andhra Pradesh, India

**Keywords:** Groundwater, Managed aquifer rechargers, Machine learning, Prediction, Sustainability

## Abstract

Managed aquifer recharge (MAR) replenishes groundwater by artificially entering water into subsurface aquifers. This technology improves water storage, reduces over-extraction, and ensures water security in water-scarce or variable environments. MAR systems are complex, encompassing various components such as water storage, soil, meteorological factors, groundwater management (GWM), and receiving bodies. Over the past decade, the utilization of machine learning (ML) methodologies for MAR modeling and prediction has increased significantly. This review evaluates all supervised, semi-supervised, unsupervised, and ensemble ML models employed to predict MAR factors and parameters, rendering it the most comprehensive contemporary review on this subject. This study presents a concise and integrated overview of MAR’s most effective ML approaches, focusing on design, suitability for water quality (WQ) applications, and GWM. The paper examines performance measures, input specifications, and the variety of ML functions employed in GWM, and highlights prospects. It also offers suggestions for utilizing ML in MAR, addressing issues related to physical aspects, technical advancements, and case studies. Additionally, previous research on ML-based data-driven and soft sensing techniques for MAR is critically evaluated. The study concludes that integrating ML into MAR systems holds significant promise for optimizing WQ management and enhancing the efficiency of groundwater replenishment strategies.

**Supplementary Information:**

The online version contains supplementary material available at 10.1007/s11356-024-35529-3.

## Introduction

Water is a fundamental and finite resource essential for life, ecosystems, and human activities. Effective water resource management is critical due to the growing pressures from population growth, climate change, and industrial demands (Martínez-Valderrama et al. 2023). Access to clean and safe water supports human survival by meeting basic needs such as drinking and cooking and is crucial for agriculture, which plays a key role in global food security. Sustainable water management practices, including efficient irrigation systems and conservation efforts, are vital for productive agriculture and environmental protection. Moreover, industries and energy production heavily rely on water, making sustainable management essential for economic development and resilience in the face of climate change (Salem et al. 2022). The interplay between water management and groundwater use is complex. Groundwater is a crucial backup during droughts or when surface water sources are insufficient. However, if not managed properly, excessive dependence on groundwater can lead to its depletion and other adverse effects (Guo et al. 2023). Therefore, balancing surface water and groundwater use is essential to maintain ecosystem health and ensure long-term water availability.

Managed aquifer recharge (MAR) projects offer several benefits for sustainable water management by addressing various water-related challenges (Escalante et al. 2023). These projects play a crucial role in enhancing groundwater storage and replenishment, providing a reliable water source during periods of scarcity. MAR supports drought mitigation by increasing groundwater resources and acting as a buffer when surface water availability is low (Yuan et al. 2023). It also helps prevent land subsidence in areas with excessive groundwater extraction and improves water quality by recharging aquifers with treated surface water or rainwater. By reducing reliance on surface water, MAR supports groundwater-dependent ecosystems, enables the reuse of treated wastewater, and enhances resilience to climate change, thereby creating a more robust water supply system. MAR techniques involve infiltrating water from various sources into aquifers and extracting the treated water for future use. Methods include aquifer storage and recovery, soil aquifer treatment, percolation tanks, bank filtration, infiltration basins, and subsurface dams (Page et al. 2023). However, the implementation of MAR projects comes with significant cost implications influenced by factors such as project scale, location, geological conditions, and chosen techniques. These costs include infrastructure for recharge basins and wells, treatment of wastewater, land acquisition and preparation, and ongoing expenses for operation, maintenance, and monitoring. Additionally, community engagement, regulatory compliance, and research add to the overall costs. Despite these substantial initial investments, the long-term benefits of MAR such as a sustainable water supply and enhanced resilience to climate variability—often justify the expenditure. Identifying suitable sites for MAR projects presents challenges related to geological, hydrogeological, environmental, and socio-economic factors. Key challenges include complex hydrogeological conditions, the availability of reliable data on hydrogeological and climatic factors, poor water quality in potential recharge sources, community acceptance, and the impact of climate variability on recharge rates (Alam et al. 2020; Khan et al. 2020; Varouchakis et al. 2023; Zhiteneva et al. 2023). Effective strategies for MAR implementation include assessing aquifer characteristics and permeability, understanding groundwater flow and precipitation patterns, using GIS tools for site selection, and conducting pilot projects to test feasibility.

Machine learning (ML) can significantly improve the selection of MAR sites and enhance the effectiveness of MAR techniques by leveraging advanced tools for data analysis, prediction, optimization, and decision-making. ML algorithms offer the ability to analyze and interpret complex hydrogeological data, predict water quality (Goralski and Tan 2020; Shiri et al. 2021; Gholami et al. 2022), and forecast future precipitation patterns using climate data (Bagheri et al. 2017). Additionally, ML can evaluate the economic viability of MAR projects and contribute to data integration by managing large and diverse datasets. Despite these advantages, practical challenges such as insufficient input data and lengthy modeling times persist (Li and Hsu 2020; Pal and Chakrabarty 2020; Mosaffa et al. 2021). Addressing these challenges is essential for fully realizing the potential of ML in optimizing MAR implementations. Integrating machine learning (ML) with alternative technologies for MAR enhances system performance and data analysis. Remote sensing via satellites and drones provides large-scale and high-resolution data, while geophysical methods like electrical resistivity tomography (ERT) and ground-penetrating radar (GPR) offer detailed subsurface insights. Data-driven and economic models benefit from ML’s predictive capabilities, optimizing recharge strategies and project planning. Smart metering and IoT devices supply real-time data for improved monitoring, while environmental DNA (eDNA) offers additional water quality assessment. Model-data fusion, citizen science, and soft sensors (Wang et al. 2021; Perera et al. 2023) further enhance monitoring and decision-making. This integration enables comprehensive analysis, accurate predictions, and efficient groundwater management. The sensors used in MAR are often exposed to harsh environmental conditions, such as high temperature, humidity, and corrosion, which can cause fouling or damage (Levintal et al. 2023; Upwanshi et al. 2023). These conditions can lead to a decrease in the accuracy and reliability of the process performance, ultimately affecting system performance (Wang et al. 2021). In control systems, a failure of a sensor can result in significant damage, making it essential to use closed-loop sensors to improve the probability of cascade error propagation. To guarantee secure and dependable functioning, it is crucial to identify, analyze, and predict errors at an early stage. Hence, integrating machine learning (ML) and deep learning (DL) models into the development of soft-sensors can function as a substitute for commonly used hard-sensors and offer a valuable resolution (Perera et al. 2023).

**Fig. 1:**
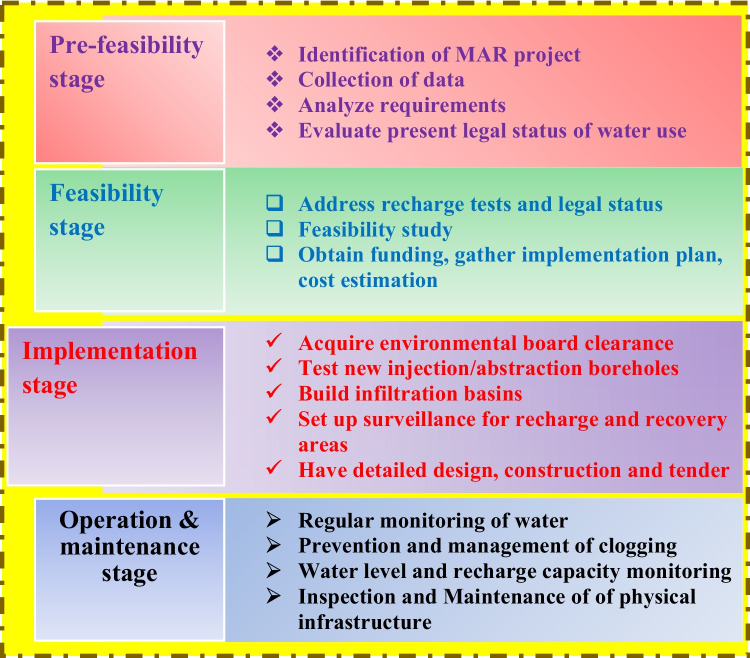
Different stages for effective MAR schemes

There is limited availability of comprehensive assessment studies regarding the utilization of ML for predicting groundwater levels in managed aquifer recharge (MAR) systems (Rajaee et al. 2019; Tao et al. 2022). This article aims to address the existing literature gap by exploring the emergence and application of various machine learning (ML) models for groundwater management (GWM) in MAR projects. The study also provides a concise literature review of historical patterns, problematic aspects, applications, and future potential uses of ML models in MAR, offering insights into how these technologies can advance GWM practices. This review article is structured as follows for the remaining portions: the review’s body introduces the literature review of analysis of article keywords and current developments in MAR. Applications and attributes of the ML models are elucidated in Section 3. Furthermore, discussions on the usage of ML in smart groundwater management of MAR are also presented in this study (Fig. 1). A case study illustrating the implementation of ML in MAR is provided as a reference to support and validate. The paper’s topics’ obstacles and unexplored study directions are elucidated, and followed by the development tools for putting ML into practice are offered. The conclusion of this novel concept is put forward along with a few unsolved research issues.

### Analysis of article keywords and current developments

**Fig. 2:**
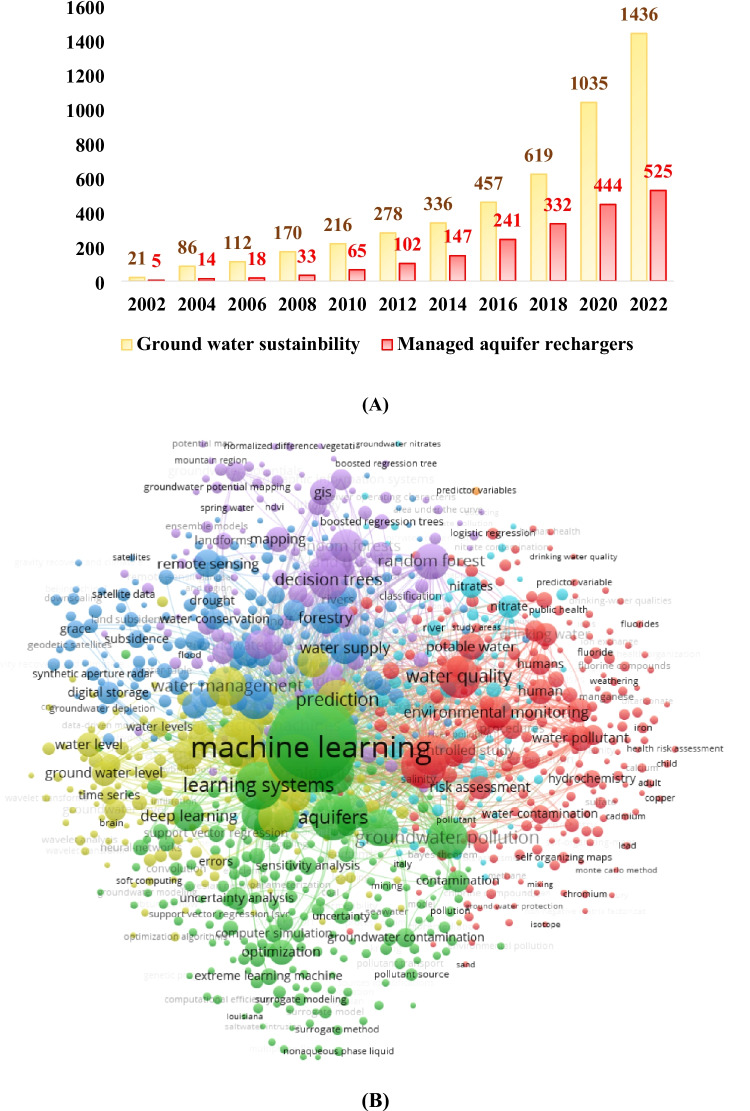
**A** Research trends integrating AI in MAR of keyword-groundwater sustainability

Figure 2(A) presents the number of articles published on the utilization of ML in MAR, along with the total count of publications. All the data and details in Fig. 2(A) are derived from keyword searches conducted on the Web of Science. Notably, a limited but precise body of literature focusing on the intersection of ML and MAR is identified through keyword searches in scientific databases like IEEE Xplore, ACM Digital Library, ScienceDirect, and Google Scholar. It is crucial to note that research in these domains is continually evolving, and new articles may have emerged since 2010 (Stefan and Ansems 2018). MAR, defined as the active replenishment of aquifers with Stormwater Flood (SF) or treated wastewater, employs various techniques like infiltration basins, injection wells, or spreading grounds. This method entails the controlled introduction of water into aquifers, aiming to enhance groundwater supplies. The primary objectives of MAR systems include improving water availability, addressing water scarcity issues, and mitigating the adverse impacts of excessive pumping and drought. The International Groundwater Resources Assessment Centre (IGRAC) is currently working on establishing a global inventory of MAR locations, facilitating locally based projects by identifying geographically and typologically proximate MAR sites (Zhang et al. 2020). The incorporation of innovative approaches such as ML and big data analytics in MAR is a relatively recent development and should be promoted. This encourages the generation of evidence-based outcomes, fostering reliable planning and implementation of MAR strategies.

These sorts of evaluations inform academics about how earlier studies evolved through time and where they may go in the future. Many software packages are available for bibliometric analysis (Kemeç and Altınay 2023). However, VOSviewer software is one of the best options for creating network maps and data. Nees van Eck and Ludo Waltman (2009) created this software program. Co-occurrence analysis is done using the software to find the most commonly used keywords in the article’s title, author’s keywords, and abstract to produce a map that connects keywords used in the paper. The number of publications containing these two keywords determines the strength of this association. From the Scopus database searching words such as groundwater, machine learning, and aquifers, a total number of 1436 documents were found from 2014 to 2024. Figure 2(B) depicts the relationships between the groundwater sector’s ML and GWM/aquifers systems. The map, created using VOSviewer software, includes four distinct clusters representing specific research areas: AI and ML applications in water quality and assessment (red), groundwater levels (yellow), decision tree models (violet), and optimization (red).

### Applications and attributes of the ML models

**Fig. 3:**
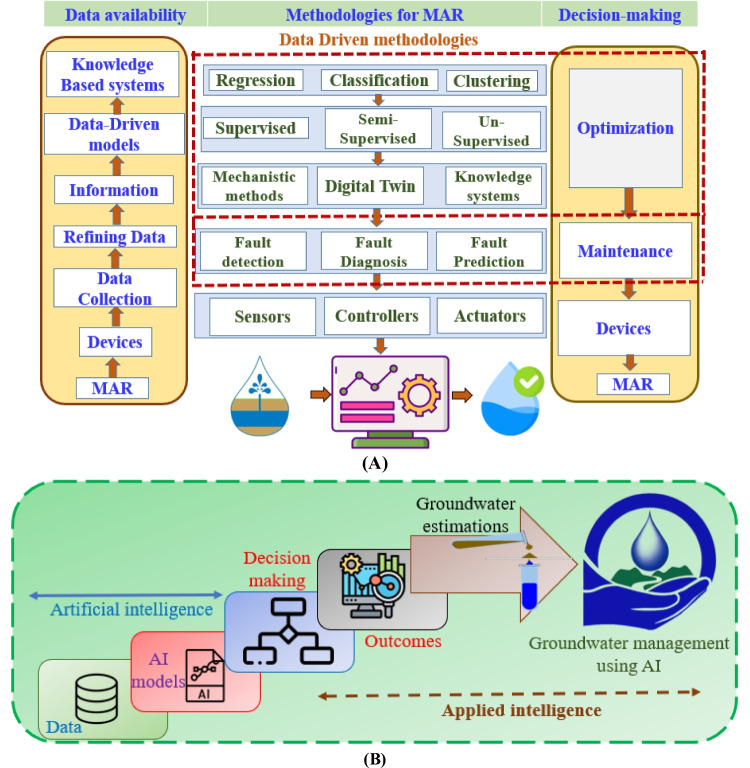
**A** Data-driven methodologies, and fault management in the MAR. **B** Potential applications of AI in GWM

A data-driven method analyzes historical datasets collected from a system and uses advanced statistical models (ASM) or ML to discover patterns hidden in the datasets. By analyzing data and assessing faults, ML techniques can build a complete fault management platform (Zhou et al. 2021). As illustrated in Fig. 3(A), a data-driven fault management platform has three main components: data availability, data-driven methodologies, and decision-making. A data-driven solution can be classified into a couple of categories, depending on the input–output (X–Y) data sets available: (a) supervised methods, such as classification and regression, which learn from pairs of X–Y and look for differences between them; (b) In unsupervised learning, a set of X without Y is used to find similarity (e.g., clustering); (c) In semisupervised learning, a set of X with a partial set of Y is used to find similarity (e.g., classification, and regression) (Liu and Xie 2020). For fault identification, it is common practice to use dimensionality reduction techniques such as principal component analysis (PCA), factor analysis (FA), independent component analysis (ICA) (Zhou et al. 2016), or its kernel variations (Hanoon et al. 2021). They can lessen the dimensionality of the monitored data, boosting disparities between various data groups and allowing for the identification of inaccurate samples. To determine a new sample corresponding to one of the normal classes or one of the aberrant classes, classification is a form of ML algorithm that can be used for fault diagnosis (Hanoon et al. 2021). Data dimensionality reduction techniques are particularly useful for fault identification because they help address several challenges commonly encountered in fault detection and diagnosis, such as high-dimensional data, noise, and the need for efficient real-time monitoring (Guo et al. 2019). Dimensionality reduction techniques like PCA or linear discriminant analysis (LDA) reduce the number of variables while retaining the most relevant information for fault identification (Ullah et al., 2024). This simplifies the analysis and allows algorithms to focus on the most critical features. For example, in a system with hundreds of sensors, PCA can identify a smaller number of “principal components” that explain most of the variation in the data, significantly reducing the complexity of fault identification models. Support vector machine (SVM), neural network (NN), and relevance vector machine (RVM) are the methods that are most frequently utilized. One-class classifiers are typically employed in classifiers to detect a single error, whereas multiple-class classifiers can automatically detect numerous problems. Prediction is the main application of regression. For example, fault evolution can be predicted using multi-step ahead prediction aiding with regressions (Goralski and Tan 2020; Che Nordin et al. 2021). It is necessary and expected to experiment and discover which algorithm and configuration will maximize performance for a specific task even though a generic decision to choose the best algorithm for fault detection, diagnosis, or prognosis is very difficult. This model follows the same process that the human brain does. Similarly, neurons make use of past information to improve feedback in the future.

To perform computational processing of neurons in an ANN model, the use of transfer function (TF) is necessary. To produce results, it is performed to the weighted sum of the input (Patel and Modi 2020; Poursaeid et al. 2022). Using TF, the hidden layer generates intermediate output, which is subsequently transferred to the associated nodes in the following level until the output level is identified. TF and its associated neurons in multiple layers are utilized to train networks. These functions facilitate the identification of sequences, modeling of processes within allowable limits, and matching of data with the original batch process data. Several nonlinear functions are used to achieve the desired output. A bias is applied to the model to achieve the best fit after calculating the weighted sum of the model. Input and output layers are mapped non-linearly using the activation function (AF) (Ostad-Ali-Askari et al. 2017). There are some characteristics of every AF. For example, sigmoidal AF allow a network to be mapped more quickly, but logistic sigmoid AF causes a network to become stuck at a local minimum or maximum throughout training. The selection of the TF’s and AF’s are very crucial to evaluating the process performance of the algorithms. To evaluate the performance of ML, the metrics used are accuracy, precision, recall, F1, mean square error (MSE), root mean square error (RMSE), sum of squared errors (SSE), mean absolute error (MAE), and R2. Among these metrics, R2 is the most widely used index, with values ranging from 0 to 1. Values closer to 1 indicate better predictive results, while values closer to 0 indicate poorer performance (Che Nordin et al. 2021; Hanoon et al. 2021; Gholami et al. 2022; Siabi et al. 2022). Additional metrics, such as training and testing accuracy of unseen data, Cohen’s kappa score, and Matthew correlation coefficient (MCC), are available to verify whether the models are truly generalizing or are just guessing or memorization (Li et al. 2023; Chicco and Jurman 2020). Table 1 reports the most frequently used functions. In supplementary data, Table S1 reports the ML performance metrics on the application of GWM.

**Table 1:** Most frequently used functions on the application of GWM for MAR

Activation/transfer	Referred Eq	Range of output	Citation
Linear	$\mathrm{f}\left({\mathrm{x}}_{\mathrm{i}}\right)={\mathrm{x}}_{\mathrm{i}}$	−	(Shiri et al. 2021; Siabi et al. 2022)
Sigmoid	$\mathrm{f}\left({\mathrm{x}}_{\mathrm{i}}\right)=\frac{1}{1+{e}^{-{x}_{i}}}$	A value between 0 and 1 can be mapped to the input using this function	(Ostad-Ali-Askari et al. 2017)
Tangent-hyperbolic	$\mathrm{f}\left({\mathrm{x}}_{\mathrm{i}}\right)=\mathrm{tan}\left({x}_{i}\right)$	One could convert the input into any number between 0 and 1 (except those values)	(Seo et al. 2022)
Gaussian	$\mathrm{f}\left({\mathrm{x}}_{\mathrm{i}}\right)={e}^{{-{x}_{i}}^{2}}$	The Gaussian function produces the same result for both negative, and positive inputs because it has an even function	(Starn and Belitz 2018)
Step Ramp	$$f(x)=\left\{\begin{array}{c}0:{x}_{i}\le T\\ 0:{x}_{i}>T\end{array}\right.$$ $$f\left(x\right)=\left\{\begin{array}{c}0:i<T\\ \frac{\left({x}_{i}-{T}_{i}\right)}{\left({T}_{2}-{T}_{1}\right)}:{T}_{1}\\ 1:{x}_{i}>T\end{array}\right.\le {x}_{i}\le T2$$	−	(Ostad-Ali-Askari et al. 2017) (Gunnink et al. 2021)
Rectified linear	$\mathrm{f}\left({\mathrm{x}}_{\mathrm{i}}\right)=max\left(0,x\right)$	−	(Kumari et al. 2023)
Pure linear	$\mathrm{f}\left(\mathrm{z}\right)=z$	$-\infty\;to\;\infty$	(Ahmadi et al. 2022)
Logistic sigmoid	$\mathrm{f}\left(\mathrm{z}\right)=\frac{1}{1+{e}^{-z}}$	0 to 1	(Sahoo 2022)
Hyperbolic tangent sigmoid	$\mathrm{f}\left(\mathrm{z}\right)=\frac{{e}^{2x}-1}{{e}^{2x}+1}$	− 1 to 1	(Che Nordin et al. 2021)

### Use of ML in smart groundwater management of MAR

Humanity must harness technology and ML innovations to meet short-term economic needs while concurrently preserving groundwater aquifers and ensuring bioregional sustainability for the future (Salehnia et al. 2019). Despite minimal changes in water supplies and sources, management tools have undergone significant advancements. The implementation of ML-driven smart groundwater management (SGWM) is proving beneficial for water utilities. ML’s advancements enable more effective and cost-efficient monitoring of water quality (WQ) in MAR, ensuring public health (Masciopinto et al. 2017). Novel ML tools emulate human learning processes, employing expert systems or rule-based algorithms for just-in-time applications or analyzing alternative options (Pourghasemi et al. 2020). In the ever-changing environment of water resource management (WRM), ML emerges as an ideal tool due to its adaptability and efficient processing of vast volumes of real-time data. This allows water utility managers to optimize current revenues while strategically planning. Innovative software-as-a-service platforms, coupled with affordable sensors and communication networks, empower water utilities to enhance productivity, cost-effectiveness, and dynamic strategic accounting activities. ML’s transformative capabilities empower water professionals, fostering imagination and paving the way for innovative solutions (Rajaee et al. 2019; Che Nordin et al. 2021). The intelligent use of ML enables managers to maximize their decisions and infrastructure investments by combining predictions with groundwater recharge forecasts and infrastructure maintenance assessments. However, the effectiveness of ML is contingent on the quality of data input and managerial understanding of the output. In the short term, human interpretation remains essential, but as ML evolves to become more sophisticated and intelligent, human interaction will diminish, making the tipping point more evident. Ultimately, the objective of machine learning is to surpass human performance, rather than achieving perfection (Al-Adhaileh et al. 2022). Figure 3(B) illustrates the diverse applications of ML in groundwater management.

#### Groundwater prediction for implementing MAR using ML

**Fig. 4:**
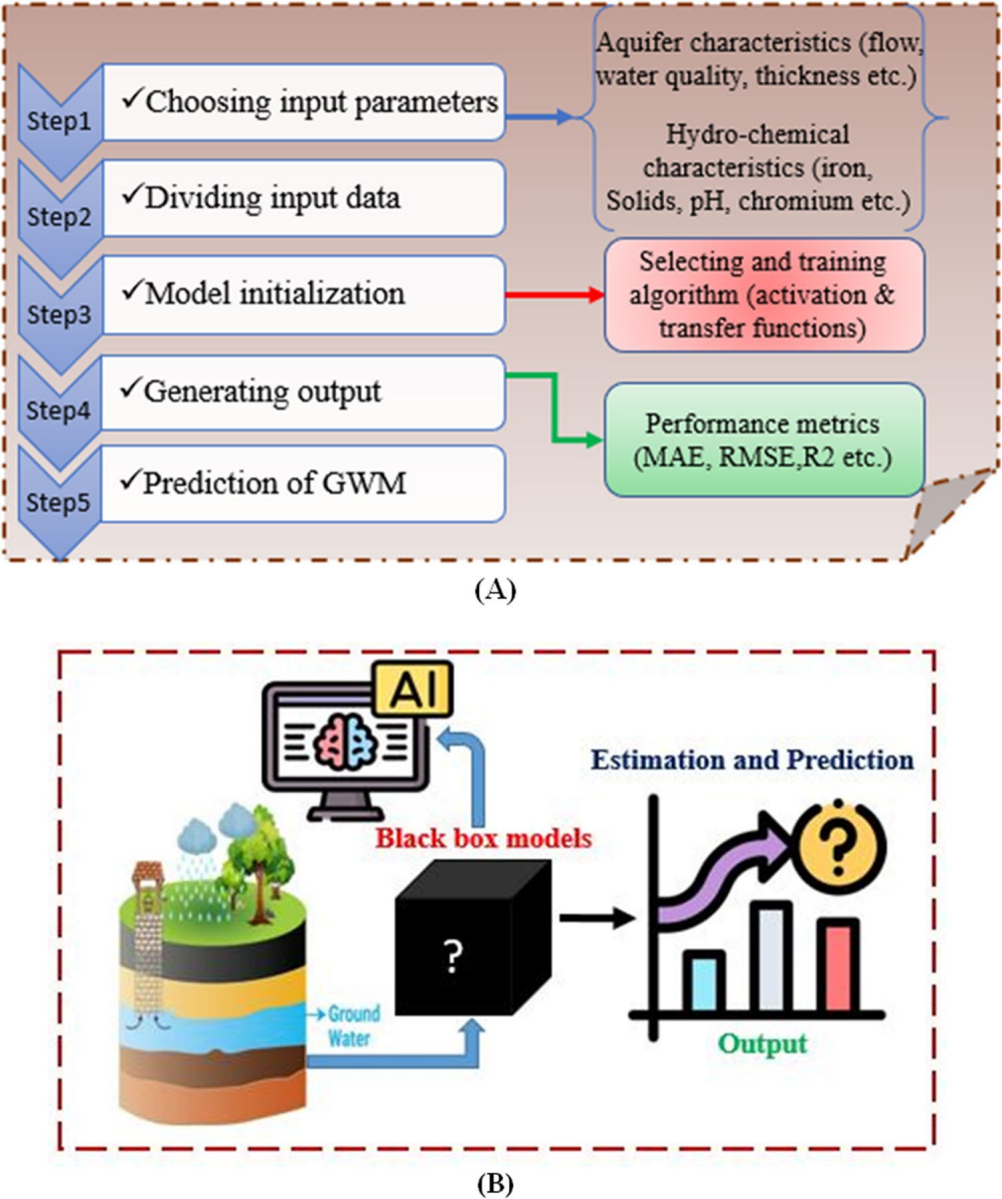
**A** Step-by-step procedure for executing the NN algorithm. **B** Groundwater monitoring using AI (black box models) in MAR

Groundwater (GW) modeling stands out as a crucial topic in contemporary hydrology, providing decision-makers with insights into water balance situations (Milan et al. 2018). Traditional GW resource modeling, relying on conventional models, has faced limitations in practicality and temporal scope (Milan et al. 2018). The need for precise data, often unavailable in many regions, adds to the constraints of these models, incapable of accommodating the non-linear and non-stationary characteristics of data structures. To overcome these limitations, various ML methods, particularly in areas with limited or inaccurate data, have become increasingly valuable (Moghaddam et al. 2019; Nguyen et al. 2020). Among the sophisticated ML models, artificial neural network (ANN) models created for groundwater-level modeling have proven effective, showcasing competence in handling non-linear and non-stationary challenges without relying on complex formulas or correlations (Ahmadi et al. 2022). The popularity of feedforward neural networks (FFNN), a type of ANN, endures due to their accuracy in GW resource studies (Ahmadi et al. 2022). The optimization of ANN using cutting-edge techniques such as particle swarm optimization (PS) and Whale Optimization Algorithm (WO) has been explored for enhanced accuracy in predicting regional GW levels (Kayhomayoon et al. 2021b). Studies have demonstrated the successful application of ML and deep learning (DL) in predicting GW levels across diverse geographical setups (Moghaddam et al. 2019; Kayhomayoon et al. 2021a). The effectiveness of ML models, particularly ANN, is heavily dependent on the quality of input data. Despite various ML models being compared, ANN, and specifically FFNN, maintains its popularity and accuracy in predicting GW levels (Ahmadi et al. 2022). Optimizing ANN using advanced techniques, like particle swarm optimization (PS) and Whale Optimization Algorithm (WO), has proven to enhance simulation accuracy (Kayhomayoon et al. 2021b). The application of ML and DL in predicting GW storage capacity, considering factors like Harris Hawks optimization (HHO)-Adaptive Neuro-Fuzzy Inference System (ANFIS) and Least Squares Support Vector Machines (LS-SVM), has been explored (Kayhomayoon et al. 2021b). Studies predicting GW levels in various regions, such as China, South Africa, and Iran, have employed ML models like Nonlinear Autoregressive Exogenous Neural Network (NARXNN), Neural Network Autoregression (NNAR), Support Vector Regression (SVR), and Hybrid SVR optimized with Particle Swarm (SVR-PS). Studies also show the effectiveness of these models in surpassing traditional methods and the importance of optimization strategies in enhancing accuracy (Di Nunno and Granata 2020; Gibson 2020; Mozaffari et al. 2022; Pham et al. 2022; Gaffoor et al. 2022). The use of ML in GWM and MAR has become indispensable for better delineation of suitable areas in semi-arid zones. Raheja et al. (2024) conducted a comparative analysis of gradient-boosting algorithms for predicting groundwater quality, concluding that the Gaussian process boost (GPBoost) with random search had the highest efficacy. Iqbal et al. (2021) constructed an ensemble model that integrates boosting and bagging algorithms for the prediction of groundwater levels, with elevated accuracy. Naganna et al. (2020) assessed gradient tree boosting (GTB) for groundwater level prediction, revealing its enhanced efficacy relative to ANFIS and Group Method of Data Handling (GMDH) models. LSTM models have predicted groundwater levels in several places using precipitation, temperature, and teleconnection patterns (Robinson et al. 2024). Continuous wavelet transform analysis and LSTM frameworks can help predict groundwater response to precipitation. Additionally, integrating LSTM with partial mutual information and bootstrap methods has increased groundwater level forecasting accuracy and reduced uncertainty across climate zones (Chu et al. 2022). However, despite the wealth of ML applications in GWM, there is a notable gap in studies exploring the effectiveness of deep neural networks (DNN), interpretable artificial intelligence (XAI), fault detection analysis (FDA), and ML and DL in GWM in MAR predictions (Mojtaba Zaresefat et al. 2023; Salehi Shafa et al. 2023. Table 2 provides an overview of the state of the art in GW modeling using ML, highlighting its significance in identifying appropriate locations for MAR. Figure 4(A) depicts a step-by-step procedure for executing the neural networks (NN) algorithm, (B) groundwater monitoring using AI (black box models) in MAR.

**Table 2:** State of the art in the modeling of GW in aquifer rechargers by using ML for water quality prediction

No	ML algorithms	Variables used	Performance metric	Remarks	Reference
1	BN, and BRT	The 41 variables are used which include soil, water, and the number of model data	MSE, R^2^	The data set appears to be a good fit for tree approaches because they are adaptable, robust to outliers, able to tolerate irrelevant inputs, and versatile. If sensoring is applied at higher levels, random forest classifications or probabilistic BRTs may be more adaptable	(Nolan et al. 2015)
2	ANN	pH, TDS (total dissolved solids), metal ions (Ca^2+^, Mg^2+^), sodium (Na), sulphates (SO_4_), potassium (K), chlorin (Cl), carbonates, nitrates (NO_3_), and %Na (sodium)	MARE, *R*^2^, & RMSE	It has been noted that there is a good correlation between the groundwater irrigation appropriateness indices produced by ANNs and the real data for both training and testing datasets	(Wagh et al. 2016)
3	SVM	Temperature (T), groundwater depth, TDS, pH, land use, dissolved oxygen (DO), and NO_3_	R^2^, RMSE	It was easy and simple to combine the SVM model’s output with GIS interpolation techniques to create a map of the examined area's nitrate level that was in good agreement with the output of the transport models	(Arabgol et al. 2016)
4	GRNN, MLP, and RBFNN	Metal ions, Na, SO_4,_ K, & Cl	MARE, *R*^2^, & RMSE	The salinity predictions from MLP, RBFNN, and GRNN were integrated by the committee neural network (CNN). Each forecast had a weight factor indicating how much it contributed to the final prediction. The salinity forecasting outcomes produced from CNN indicated that the CNN outperforms each individual ANN functioning alone to anticipate groundwater salinity	(Barzegar et al. 2017)
5	ANN	Heavy metals	*R*^2^, & RMSE	Based on the outcomes of the simulation, it was shown that ANN could be used to accurately anticipate the concentration of heavy metals in groundwater resources	(Alizamir and Sobhanardakani 2016)
6	ANN	pH, electrical conductivity (EC), & total hardness (TH)/Na	*R*^2^, & RMSE	Three characteristics, pH, EC, and TH, were determined to be the optimal input factors by pre-modeling. The outcomes showed that training six monitoring wells to a high degree of precision can precisely determine the Na content in three wells	(Heidarzadeh 2017)
7	ANN-diffFadipeFadipetial evolution (DE), & ANN-particle swarm optimization (PSO)	EC, metal ions, pH, TDS, SO_4_, K, Cl, & Na	*R*^2^, RMSE & MSE	Both ANN-PSO and ANN-DE are suitable methodologies for modeling groundwater quality, according to the study's results and findings. However, it can be seen that the DE-based model performs better than the PSO-based model during the training, validation, and test phases	(Kisi et al. 2017)
8	ANN & wavelet neural network (WNN)	TDS, COD, Cl, SO_4_, NO_3_/ WQ grade	*R*^2^, RMSE, & MAE	The outcomes of the three techniques show that WNN is more accurate than the other two techniques in the manuscript. The study shows that these techniques are effective tools for determining the quality of GW	(Yang et al. 2017)
9	ANN & SVM	Metal ions, Na, SO_4_, K, Cl, GW level, EC, & pH	*R*^2^ & RMSE	ANNs and SVMs gave more precise estimations of the three qualitative variables as compared to cokriging interpolation approach, according to these RMSE values. *p*-factor and *d*-factor were used to calculate the output uncertainty of ANNs and SVMs. The findings proved that SVMs are less uncertain than ANNs	(Isazadeh et al. 2017)
10	ANN	pH, Cl, SO_4_, & TDS	*R*^2^	The findings produced with the use of this ANNM model exhibited great prediction efficiency, as the SSQ functions and R2 were determined to be (0.038 and 0.005) and 0.973, respectively. However, the parameters pH and Cl had a considerable impact on model prediction, making them important variables in the anticipation performed using the ANN model	(Khudair et al. 2018)
11	ANN (MLP (mutilpe layer perceptron-NN (newral network), radial basis function (RBF)-NN)	pH, EC, TDS, metal ions, abstraction rate/NO_3_	*R*^2^, RMSE, & MAE	The MLP-NN’s prediction outcomes have been found to be superior to RBF. The outcomes of the predictions illustrate the NN approach’s good and broad application for predicting nitrate in the GW wells of the coastal aquifer in the Gaza Strip	(Zaqoot et al. 2018)
12	Adaptive neuro-fuzzy inference systems (ANFIS)	pH, SO_4_, Na, K, Cl, Hardness, EC, metal ion, HCO_3_/NO_3_	*R*^2^, & RMSE	The ANFIS model V, out of the five models created, predicts nitrate content with 90% accuracy and a smaller RMSE (0.0934). When predicting the spatial distribution of GW nitrate content, GIS uses the model V results as input	(Jebastina and Prince Arulraj 2018)
13	MLP, gene expression programming (GEP), SVM, ANFIS	Na, HCO_3_, metal ions, and SO_4_/TDS	*R*^2^, RMSE, & MAE	The findings showed that the estimation of TDS alterations could be accomplished using the AI models. By conducting a comparison among various soft computing methodologies, it was established that the GEP model outperforms the MLP, SVM, and ANFIS models, thus affirming its superiority	(Jafari et al. 2019)
14	ANN & MLR (multiple linear regression model)	Metal ions, Na, SO_4_, K, Cl, GW level, EC, & pH	*R*^2^	To evaluate the effectiveness of ANN prediction, MLR model is utilized. The outcomes verified that the ANN model’s predictions are accurate and show its consistently respectable performance for both seasons. Similar investigations of GW quality prediction for drinking purposes may benefit from the proposed ANN model	(Kadam et al. 2019)
15	SVM-Kernel-NN, RF	Elevation, distance from stream, hydraulic conductivity, land use/NO_3_	*R*^2^ & RMSE	The RF model demonstrated slightly superior prediction performance compared to the kNN model, it was found to be less reliable. Considering both predictive accuracy and the level of uncertainty, the KNN model emerges as the relatively better option for forecasting nitrate levels in groundwater	(Rahmati et al. 2019)
16	DL & ANN- principal metal index (PMI)	Heavy metals, & Zn pollution indices	RMSE, MSE, & MAE	The highest R2 values were achieved by the prediction accuracy of PMI using both ANN and DL models, which were 0.95 and 0.99, respectively. The proposed DL model presents a suitable approach in the realm of computational chemistry, particularly for mitigating overfitting concerns. Notably, it outperformed the conventional ANN, demonstrating its efficacy and potential superiority in this domain	(Singha et al. 2020)
17	ANN, extreme gradiant booster (XGB), & SVM	Multiple hydrogeologic, water quality, and land use/levels of NO_3_ and pesticide	NA	Other methods of predicting the quality of the water can be effectively replaced by XGB. Numerous solutions to this issue were also investigated because the WQ data is typically unbalanced. The findings reveal that most models’ prediction abilities were enhanced and their robustness increased as a result of the mitigating measures	(Bedi et al. 2020)
18	ANFIS, MLR, & SEM (structural equation modeling)	TH, SO_4_, Cl, metal ions, NO3, K, and Na/TDS	RMSE	an ANFIS model that outperforms the MLR model, supporting the findings of several previous research that claim ANFIS is superior to MLR. An MLR model is significant because it quickly evaluates WQ data and is appropriate for studies with small sample sizes	(Kumar et al. 2020)
19	ANN	Time, location, pH, TDS, SO4, Cl, NO/TH	RMSE, *R*	The interpolation maps and findings of the sensitivity analysis indicate that time and space are key factors in determining which regions have acceptable and desirable overall hardness concentration. The overall hardness concentration for all the spots could be predicted by the neural model created in this study. This study provides interpolation maps for total GW hardness, which aid in identifying areas with varying GW quality	(Sunayana et al. 2020)
20	RF	TDS, metal ions, Cl, SO_4_, K, Na, NO_3_/GW quality	MSE	The RF technique is preferable to other approaches due to its high prediction accuracy, capacity to understand nonlinear correlations, and ability to identify key prediction factors. Overall, the findings of this study proved that the RF approach might be used as a trustworthy way of assessing GW susceptibility and correctly managing or monitoring aquifers	(Norouzi and Moghaddam 2020)
21	ANN	pH, TDS, turbidity, and EC/*E. coli*	*R*^2^ & MSE	The technique produces a trained neural network by superimposing it on various neural network architectures. In order to reduce mistake, real-time bacterial monitoring must be automated in addition to producing precise data	(Khan et al. 2020)
22	Adaboost, ANN, SVR, & RF	EC, T, PH/TDS, MAR	RMSE, *R*	According to the outcomes, RF and Adaboost models perform better overall in making predictions than SVR and ANN models. As opposed to according to evaluations of generalization ability and sensitivity to input factors, the ANN and SVR models exhibit improved generalizability and reduced sensitivity compared to Adaboost and RF	(El Bilali et al. 2021)
23	RF	Metal ions, soil pH, NO_3_, and SO_4_ levels, and clay /uranium	RMSE	Groundwater quality is critically dependent on recharge conditions, which has been shown by the fact that the geochemical circumstances that lead to ternary uranyl complexes inside the aquifer are partially produced by infiltration through the vadose zone	(Lopez et al. 2020)
24	ANN	Metal and heavy ions, DO, T, pH, carbonates, TDS, SO_3_ & turbidity	*R*^2^, & MSE	At 10% increases in iron, nitrate, and chloride, the ANN simulation of pH shows good agreement, and when the parameters are increased, the acidity of the water sources decreases. For every water sample taken into consideration, the model provided a reliable estimate of TDS	(Fadipe et al. 2024)
25	XGBoost, RF, ANN, and DL	pH, SO_4_, Na, K, Cl, hardness, EC, metal and heavy ion, HCO_3,_ NO_3,_ orthophosphates, TDS, TH, T, DO, time	MAPE, RSR, MSE, *R*^2^, RMSE, NSE	By repeatedly running the suggested technique on a newly randomized dataset for 10 iterations, where slight variations in performance metrics are seen, the uncertainty of the DL (deep learning) model output is cross-verified. A further indication that the DL model is the most realistic and reliable method for predicting GW quality is the input variable importance calculated by prediction models	(Singha et al. 2020)
26	ANN	T, recharge rate, life time, GW level, aquifer thikner, total rainfall, humidity, wind speed	RMSE & *R*^2^	The results indicate that the ANN-5 model, incorporating a combination of factors, exhibits exceptional accuracy in estimating final chloride levels, as evidenced by high R2 (0.977) and low RMSE (0.022) values compared to recorded data. This suggested approach showcases the potential of utilizing the ANN algorithm for modeling, specifically in identifying crucial variables that significantly influence chloride levels	(Kassem et al. 2021)
27	Boosted regression trees (BRT)	Soil contents, and aquifer material/pH	%bias, NSE (Nash–Sutcliffe efficiency), RMSE, *R*^2^	These findings indicate that empirically rooted machine learning techniques, such as BRT, can be used to predict WQ conditions in aquifer systems that are too big and stratigraphically complicated to allow for GW flow and reactive solute transport simulations in a single domain	(Stackelberg et al. 2021)
28	ANN & modular neural network (MNN)	Transmissivity, depth to the water level, topography watershed, GW recharge, precipitation, & evaporation	MAE, MSE, R, & NSE	The MNN performed best in terms of GW drawdown modeling. The annual drawdown across the entire aquifer was mapped using the best network that was fitted to the input data that was available. The selected methodology can be used to anticipate GW drawdown in the research site and other similar environments	(Gholami and Sahour 2022)
29	Improved Alpha-Guided Grey Wolf optimization (IA-GWO), PSO & ANFIS, & ANN	Groundwater level	NRMSE, NSE, relative absolute error (RAE)	NFIS-IA-GWO > ANFIS-PSO > ANN-IA-GWO > ANN-PSO > ANFIS > ANN	(Singh and Panda 2022)
30	MFT, SDF, FMF, MMA, GA GEP, MFT-MMA	gypsum, clay, hydraulic conductivity, salinity, sandstone and carbonate,	*R*^2^, RMSD, MMA, normalized AVI (N-AVI), receiver operating characteristic (ROC), and area under the curve (AUC)	The key outcome highlights the critical role that model combination and supervision play in developing an acceptable model of vulnerability boundaries even with a small dataset	(Msaddek et al. 2022)
31	FFNN, AIG, BWO	Monthly precipitation (P), T, and water table height	MAE, MSE, *R*^2^, & NSE	As compared to ANN-BWO and ANN-CSO, the hybrid ANN-AIG model was more accurate. The outcomes showed that the hybrid models performed satisfactorily in enhancing the ANN model’s capacity for estimate by 20%	(Dehghani and Torabi Poudeh 2022)
32	SES, BiLSTM, ANFIS	WQ parameters	RMSE, MSE, CC	The results are consistent with the notion that high-accuracy WQ predictions can be made using the SES-BilSTM and SES-ANFIS models, contributing to the improvement of WQ. The outcomes showed that the predictions of the SES-BiLSTM and SES-ANFIS models were precise, and that the performances of both seasons were consistent	(Al-Adhaileh et al. 2022)
33	DT, RF, XGB	EC, pH, SO_4_, NO_3_, Cl, DO, PRO, Trim, Iop, Dicl	MAPE, MSE, *R*^2^, & MAE	To predict the removal of PP by the MAR system, XGB might be the best predictive model to use	(Yaqub et al. 2022)
34	ANN, RF, SVM, Bagging	17 groundwater determining factors are used	ROC, MAE, RMSE, SE, SP, AC, PPV	ANN-Bagging achieved the highest performance on three threshold-dependent and independent evaluation criteria, although the calibration/validation data could sometimes skew its results. In terms of prediction efficiency, RF and SVM were slightly less efficient than ANN-Bagging, Bagging	(Das and Saha 2022)
35	DNN-XGB, MLR	Nitate (NO_3_), GW depth, phosphorus, distance from industries, land use topography, and exploitation from groundwater	RMSD, NSE, NRMSD	Upon evaluating the error indices and comparing the observed and predicted values, it becomes clear that the EGB model surpasses the other two methods in accurately predicting both the minimum and maximum nitrate values. This compelling evidence highlights the outstanding performance of the EGB model throughout the modeling process	(Gholami and Booij 2022)
36	ANN, FL, ANFIS, SVM	GW level	MAE, RMSE, R, & NSE	The study recommends using ANFIS in areas with no other meteorological or hydrometric data and only groundwater level data available to predict the groundwater level. In complex aquifers, groundwater management plans may benefit from the findings of this study	(Tao et al. 2022)
37	LSSVR, ANFIS, RF, GEP, HHO	9 parameters used for GFI	MAE, RMSE, & NSE	LSSVR produced the best prediction performance out of the four models. An ANFIS with poor performance was improved by using the novel HHO algorithm. The GFI prediction performance was improved with a hybrid-HHO-ANFIS	(Milan et al. 2023)
38	SVR, GA	Rainfall, soil, parameters for GW	RMSE, *R*^2^, ROC curves, MF, fitness function	According to the results, optimum kernel values in the SVR model, along with identifying the optimal features that contributed most to the modelling, were key steps toward achieving a desired level of precision while modeling	(Al-Fugara et al. 2022)
39	ANN	Land use, distance from SF, transmissivity, water quality, thickness, rainfall, slop, soil, geology	MSE, RMSE, *R*^2^	It was found that the AI technique was capable of determining the MAR sites with accuracy. In conclusion, this work uses machine learning techniques in a novel way to find the best AGR sites	(Zaresefat and Derakhshani 2023)
40	ANN, NSGA-II	GW level, TDS	R2, R, RMSE	During the study period, the total volume of ideal recharge in managed aquifer recharge (MAR) is projected to increase by 119%, while the variations in the optimal groundwater level are expected to increase by 14%. This comprehensive analysis indicates that the multi-objective modeling platform method is capable of simultaneously achieving all the goals outlined in the study	(Shafa et al. 2023)

#### Mapping and assessment for MAR

**Fig. 5:**
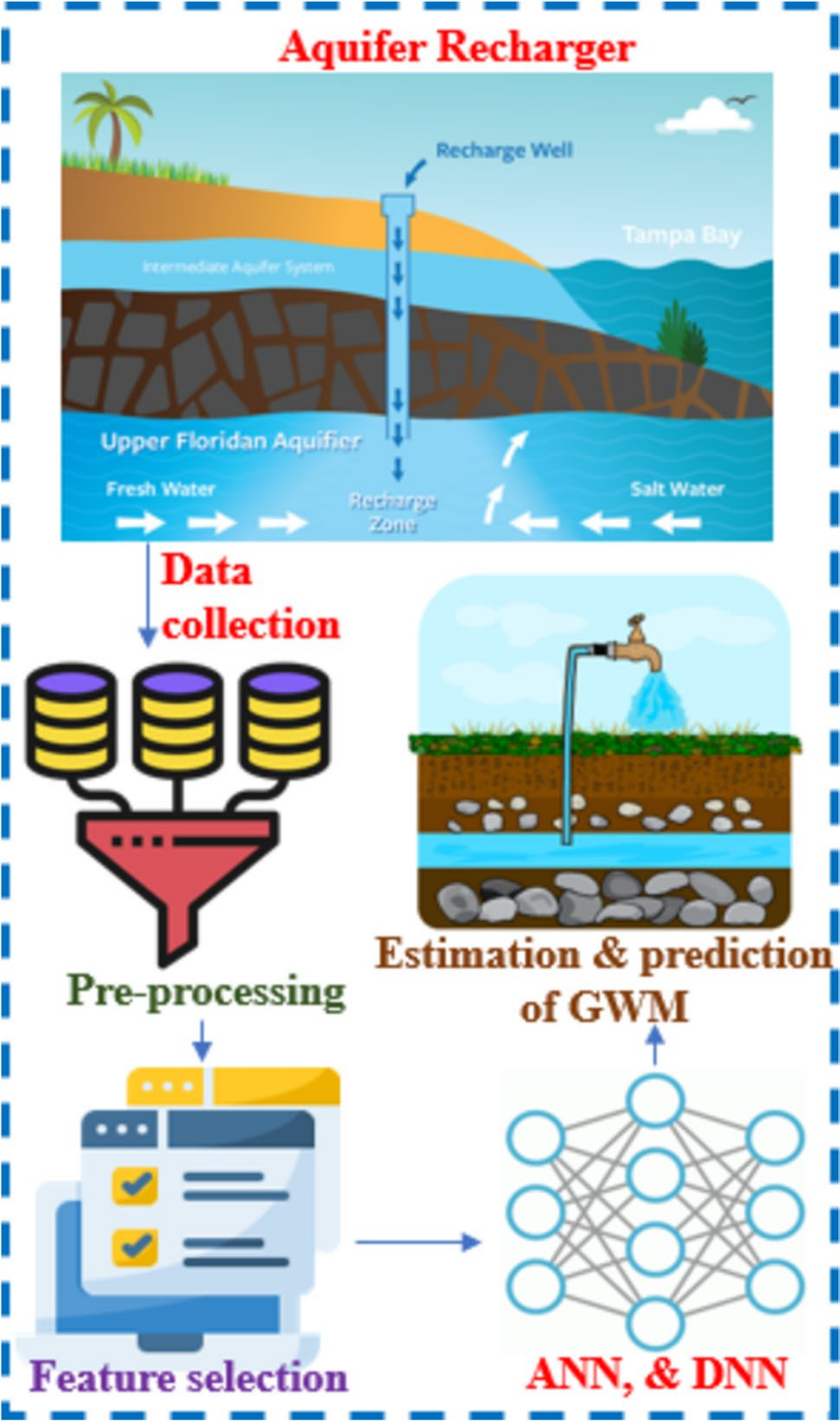
Data collection, pre-processing, feature selection, application of ANN and DNN, estimation, and prediction of GWM in MAR

Determining the suitable mapping for MAR and understanding its impact on groundwater flows and levels can pose challenges. Assessments often occur at the regional level, where detailed information on complex surface and subsurface conditions may be limited. It is crucial to consider potential variations in MAR effects based on factors such as location, size, and operational conditions. ML models, including support vector machine (SVM), artificial neural network (ANN), classification and regression tree (CRT), K-nearest neighbor (KNN), maximum entropy (ME), and functional tree boosted regression tree (BRT), among others, are employed in the mapping and assessment process (Naghibi et al. 2017; Mohsen Mousavi et al. 2017; Lee et al. 2018; Chen et al. 2019). Hybrid ML techniques, combining multiple models, are increasingly favored for assessing and mapping groundwater potential due to their enhanced performance in various scenarios compared to single ML models. Comparisons reveal that hybrid/ensemble models, such as random forest (RF), decision tree (DT), and combination frameworks of ML/DL (ABRF, BRF, and LBRF), generally outperform individual ML models in predicting potential groundwater zones. However, certain groundwater-related challenges, particularly the evaluation of GW potential in diverse locations, may not be effectively addressed by existing ML algorithms alone (Bui et al. 2020). The imperial competitive algorithm (ICA) optimization approach allowed the ANFIS model to identify the optimal parameter set and prevent any conflation of parameters essential for developing the model and training (Moghaddam et al. 2020). Consequently, cutting-edge hybrid ML techniques like LBRF are explored for their potential in groundwater potential determination across different areas (Ha et al., 2022). Al-Ruzouq et al. (2023) developed a high-efficiency artificial groundwater recharge (AGR) map for the United Arab Emirates by combining ML algorithms with geographic information systems (GIS) and remote sensing data. The RF model outperformed the others, with an overall prediction accuracy of 99%, according to the results. About 10% of the research region was classified as having high AGR potential based on the created AGR maps, which were categorized based on their eligibility for AGR potential. LSTM exhibited superior consistency in performance relative to XGBoost, attaining the maximum accuracy at three of the five locations. The models’ performance and training were adequate (Osman et al. 2024). An important consideration is the study’s principal weakness, as it overlooks subsurface geology in the model due to the predominance of sandy soil on the surface. Future studies plan to incorporate subsurface geology to enhance input parameters for the models. In conclusion, the application of ML and DL methods can significantly enhance our ability to accurately assess groundwater contributions to managed aquifer recharge. There is a notable need for ML models specifically tailored to groundwater management (GWM) potential mapping and assessment in the context of MAR. The schematic representation in Fig. 5 illustrates the stages of data collection, pre-processing, feature selection, application of ANN, DNN, and the estimation and prediction of GWM in MAR. Table 3 represents the ML applications for identifying appropriate site locations for MAR.

**Table 3:** ML applications for identifying appropriate site locations for MAR

No	ML algorithms	Variables used	Performance metric	Remarks	Reference
1	RF, BRT, SVM, mixture discriminant analysis (MDA), and multivariate adaptive regression spline (MARS)	12 hydrological-geological-physiographical (HGP) conditioning factors	Receiver operating characteristics (ROC) and area under the curve (AUC)	According to the study’s findings, the equivalent AUC values of the groundwater spring potential maps made using the MDA, RF, SVM, BRT, and MARS models were 0.832, 0.805, 0.802, 0.780, and 0.755, respectively. These area values clearly show that MDA, RF, and SVM were the top-performing models	(Al-Fugara al. 2020)
2	Adaptive neuro-fuzzy inference system (ANFIS), ANFIS-imperial competitive algorithm (ANFIS-ICA), alternating decision tree (ADT), and RF	Fifteen diverse geo-environmental factors	ARUROC	Based on performance indicators in both the training and validation stages, the RF model (AUROC = 90.74–96.32%, TSS = 0.79–0.85) was determined to be the best model. The RF method is the most effective option for geospatially modeling groundwater potential when there is a restricted quantity of observations (springs) available for predictive modeling	(Moghaddam et al. 2020)
3	RF, boosted regression tree (BRT), and the ensemble of RF and SVM	15 groundwater factors	AUROC	As per the findings obtained from RF, BRT, and RF-SVM, the research area is comprised of 33.31%, 35.60%, and 36.85% of the extremely high groundwater potential zone. The models’ predictions were verified using the AUROC curve. Models RF and RF-SVM performed better than the BRT model, according to AUROC curves	(Prasad et al. 2020)
4	Group method of data handling (GMDH), Bayesian network (BN), ANN	Groundwater level in the previous month, aquifer exploitation, surface recharge, precipitation, temperature, and evaporation	RMSE, NASH, MAPE, and *R*^2^	To improve accuracy in other aquifers, the GMDH method may eventually take the place of the ANN, which is one of the popular approaches used to estimate groundwater levels	(Moghaddam et al. 2021)
5	RF and random subspace (RSS	land use land cover (LULC), rainfall, distance to road, elevation, slope, topographic roughness index (TRI), stream power index (SPI), sediment transport index (STI), curvature, soil types, topographic wetness index (TWI)	ROC and AUC	RSS has the highest AUC for groundwater potential modeling (0.892), followed by RF (0.86). Groundwater potentiality modeling relies heavily on key parameters such as distance to river, slope, curvature, elevation, LULC, and SPI	(Sarkar et al. 2022)
6	RF, SVM, and ANN	Hydrogeological and geo-environmental conditioning variables	Area under the receiver operation characteristics (ARUROC) curve values	The ARUROC curve was used to demonstrate the precision of model prediction, with the RF model exhibiting the best prediction rate of probable zone of groundwater occurrence over the other two models	(Saha et al. 2022)
7	Frequency ratio (FR), radial basis function (RBF), index of entropy (IOE), evidential belief function (EBF), and fuzzy art map (FAM)	Number of wells (1448), flow discharge, and water table depth	ROC &AUC curves, standard errors (SE), positive predictive value (PPV), negative predictive value (NPV), sensitivity (SST), specificity (SPF), and accuracy (AC)	Among the other hybrid models, the FR-FAM and FR-RBF ensemble models show the most promise and are the most application-friendly. According to the GPMs, the northern portion of the research region has extremely high and high groundwater potential zones	(Yariyan et al. 2022)
8	SVM, multilayer perceptron, and RF	Precipitation, drainage density, TDS, GWL, geology, geomorphology, LD, elevation and distance from residences	MAE and RMSE	The results revealed that the RF model performed the best, with an overall prediction accuracy of 99%. The created artificial groundwater recharge (AGR) maps were categorized based on their AGR potential, with around 10% of the research region classified as high	(Al-Ruzouq et al. 2023)
9	Encoder-decoder, U-Net, and attention U-Net	Hypothetical artificial recharge sites (100, 300, 500, and 1000 sites	Nash–Sutcliffe efficiency (NSE)	All three ML models demonstrated minimal gains from more than 500 recharge sites, but they all improved with more training sites. As a result of their superior speed over the numerical model—with predictions within 0.24 s or the 97th percentile—the trained machine learning models are perfect for decision optimisation	(Fernandes 2024)
10	Extreme gradient boosting (XGBoost), RF, Naive Bayesian (NB), Fuzzy Multicriteria Decision Making (MCDM)	Field-based conditioning factors	ROC curves, AUCs, and correlation plots	All four models performed well overall in assessing the likelihood of groundwater occurrences. Compared to other machine learning and MCDM models, the predictive power of the XGBoost approaches with the greatest AUC values (0.79) and highest correlation value (0.78) is greater	(Halder et al. 2024)
11	Long short-term memory (LSTM), ANN, and SVR	Meteorological data of rainfall, temperature, evaporation, and groundwater level	RMSE & MAE	In terms of 1-day, 3-day, and 5-day ahead forecasts, LSTM and XGBoost significantly outperformed SVR and ANN across all locations when comparing the GWL at the wells in the identified towns predictions made using SVR and ANN. Additionally, LSTM outperformed XGBoost in more consistent ways, winning the highest accuracy in three out of the five locations	(Osman et al. 2024)

#### ML for characterizing the effectiveness of MAR

The governing equations presented by Alam et al. (2021) show the forecasting capacity of various MAR systems for penetration, storage of water for later use, and efficacy in removing contaminants. For a more profound comprehension of systems, mathematical models are frequently employed in conjunction with experiments. Explaining pollutant removal in MAR involves understanding the intricate relationships among influential soil and water characteristics. Due to its intricacy, conventional mathematical or statistical parameters struggle to accurately simulate the relationship between system properties and removal efficiency. Consequently, machine learning (ML) may provide an alternative to conventional mathematical techniques for modeling MAR. Many applications in environmental engineering have successfully utilized data-driven modeling, employing advanced optimization tools to enhance process efficiency and minimize computation costs and material requirements (Eren et al., 2019; Bhagat et al. 2020; Yaqub et al. 2021). Despite numerous publications describing the application of ML models in water treatment systems, there is a scarcity of studies focusing on creating models that forecast the success of MAR in removing pollutants for wastewater reclamation and reuse (Yaqub and Lee 2020). In their study, Yaqub and Lee (2020) utilized a multilayer perceptron neural network (MLPNN) as a black box approach with no derivable equations. In contrast, gene expression programming (GEP) allows for the creation of an expression tree (ET) used to develop a function representing the correlation between inputs and outputs. Consequently, GEP excels in predicting pollutant removal through MAR and can reduce the experimental workload. Despite the potential of “surprise” in predictive ML models, even the most advanced algorithms, mathematical frameworks, and analytical tools can face total failure under certain circumstances. Similarly, the influence of physical parameters such as soil properties, aquifer properties, climatic variation, land use and land cover, and hydro and geological conditions existing in the site (Cabalar and Akbulut 2016; Weber et al. 2017; Maples et al. 2020) is also considered. The hydraulic conductivity (HC) of the aquifer is a crucial parameter that directly affects the performance of MAR. A high HC indicates that the aquifer has good permeability, allowing water to move more easily through the subsurface. In such cases, the recharge process is more efficient, and water can infiltrate and spread within the aquifer at a faster rate. Conversely, a low HC suggests lower permeability, making it more challenging for water to move through the aquifer. In areas with low hydraulic conductivity, the recharge process may be slower, and the stored water may not be distributed effectively within the aquifer. Clogging of soil in aquifer refers to the gradual accumulation of particles or sediments in the pores caused by factors such as physical (temperature, accumulation, emulsifiers, etc.), chemical (geochemical reactions that result in the precipitation of minerals, e.g., iron, aluminum, or calcium carbonate growth, ion exchange, conductivity), biological (algae-growth, bacterial growth, biological flocs, etc.), and mechanical clogging (entrained air/gas binding, nitrogen, methane from microbiological phenomena, etc.). The HC of MAR is affected by clogging that in turn causes a reduction in porosity, permeability, change in soil structure, formation of biofilms, and accumulation of particulate matter. Thicker clogging layers are reported to be generated in systems with larger infiltration rates and smaller suspended solid sizes (Zhang et al. 2020). Physical clogging (PC) phenomena are fundamentally based on (a) filtration, (b) suspended solids, and (c) physical precipitation, where these three mechanisms play a crucial role in PC occurrences. Bouwer (2002) employed a parallel filter index in pilot plant studies to address PC, but its applicability at a large scale, especially for water pretreatment, is limited. Zheng et al. (2023) utilized COMSOL Multiphysics software for simulating and forecasting the onset and progression of PC. They reported that controlling water quality (WQ) factors responsible for clogging could be a beneficial strategy to prevent PC, making water quality control (pre-treatment) essential. Routine backwashing is commonly employed to address physical clogging issues. Chemical clogging often occurs concurrently with other types of clogging and is mainly caused by mineral precipitation at the zone where recharge water (RW) and groundwater amalgamate. Numerical modeling, including approaches like MT3DMS, PHT3D, and EASY-LEACHER, is frequently used to predict the probability of chemical blockage based on water chemistry (WC) and hydrogeological data (Ringleb et al. 2016). Biological clogging, associated with microbial communities, is addressed by eliminating organic particles from source water either before or after disinfection. Sterilization, backwashing, and well-washing are common methods to mitigate biological clogs. Mathematical and numerical models in the groundwater (GW) environment are effective tools for anticipating microbial blockage. Mechanical clogging, known as gas bubbles, can result from water cascading within recharge well casings or incoming air from the recharge piping system under negative pressure, leading to MLr bubbles that may obstruct pores. Mechanical blockage in spreading basins and recharging boreholes can be prevented by implementing backwashing and optimizing the recharge process. The promotion of big data analytics and ML technologies is crucial for producing research-backed outcomes in MAR methodology planning and implementation. While various mechanistic or automated models exist for different phases of groundwater management (GWM) in MAR, combining them may not be feasible due to variability in process parameters depending on the level of microbial activity. Although computerized systems for data recording, quality control, laboratory investigations, and knowledge-based interactions exist, the computation of inputs can differ based on microbial activity intensity (Mojtaba Zaresefat et al. 2023). The study of subsurface chemical and physical operations in MAR has significantly contributed to local problem-solving, but there is a need for further synthesis and extension to diverse physiographic settings. Tavakoli et al. (2024) tackle the issue by combining the DRASTIC index with five ML models such as RF, boosted regression trees, generalized linear model, SVM, and multivariate adaptive regression splines alongside hydrogeochemical investigation. The results provide a significant ML-based DRASTIC index to tackle salinization challenges in compromised aquifers in the Middle East and North Africa (MENA) region, emphasizing the critical necessity to mitigate groundwater pollution in swiftly urbanizing locales. Further investigation into MAR purification mechanisms is essential to determine their reliability and applicability to a broader range of circumstances and water sources. Integrating hydrogeology, ecology, and microbiology in systematic research is suggested to advance the understanding of purification phenomena in MAR. Despite the potential for prediction and control, it has been observed that very few research articles specifically highlight the use of ML and DL algorithms in the predictive control of clogging mechanisms in MAR.

#### Techniques use in MAR and ML approach

The decision-making process of MAR and its deployment under specific circumstances and demands would be aided by an understanding of the differences in MAR design and how these designs help absorb water or remove contaminants. Stefan and Ansems (2018) compiled an inventory of 1127 MAR sites based on the type of MAR, water source, purpose, and site (Stefan and Ansems 2018). It has been reported by Alam et al. (2021) that several MAR methods can be used to improve WQ, as well as improvements. Site restrictions affect the MAR-type selection. Several diverse methods are available to address the capturing and storing of water from the surface, such as infiltration basins (IB), agricultural-MAR (Ag-MAR), percolation tanks, SATS, surface flooding, and ditch-furrow irrigation systems. Due to this, MAR types of this type require large spaces without slopes. A similar area is difficult to obtain for other types of MAR when compared to AgMAR, which allows the use of large agricultural land for recharge. While Ag-MAR sites may not achieve as great a recharge rate as other techniques (e.g., infiltration basin) because of their large surface region, the net volume of water recharged is balanced (Dahlke et al. 2018; Kourakos et al. 2019). Through wells or other structures, surface water can be routed into the deep soil layer via ASR or VZIF. Therefore, they have a limited capacity for removing contaminants. The use of check dams and sand dams, as well as channel infiltration/spreading, is performed on lands with a higher slope to prevent rainwater from flowing into the surrounding soil at high levels. To remove contaminants, the filtration system causes river water to pass through the riverbank. Pumps are employed to pull water concerning the bank. The utilization of water from a non-point source in a densely populated region is the goal of stormwater BMPs, such as detention ponds, bioretention or bio-infiltration systems, and rain gardens. Consequently, these units are substantially smaller in magnitude than other MAR systems. The governing equations presented in Alam et al. (2021) can be used to forecast how well various MAR systems will penetrate, store water for later use, or remove contaminants. These equations show that site characteristics, including hydraulic conductivity, soil texture, mineral qualities, and source water chemistry, can influence the MAR’s ability to both infiltrate and remove contaminants. According to Cabalar and Akbulut (2016), soil texture affects infiltration rates, as does geologic architecture (Maples et al. 2020). MAR suitability to infiltration rate is typically assessed using the hydrologic soil group. In addition to photolysis, physicochemical filtration, precipitation, sorption, and biodegradation can also provide a means of removing pollutants from surface waters (Dominic et al. 2018). The hydraulic conductivity of the soil or the soil texture can have a direct effect on MAR’s ability to treat water, as sorption and biodegradation are dependent on hydraulic residence time. Due to changes in water chemistry, redox fluctuation, and the transformation of contaminant ushers, pollutants can desorb from the contaminated subsurface, increasing GW contamination (Weber et al. 2017). Therefore, it is crucial to select a site with soil properties that facilitate swift infiltration and storage of substantial water quantities, while also enabling the removal of pollutants from the soil through adequate residence time.

For gaining a deeper understanding of systems, mathematical models are often used in coupling with experiments. Using MAR pollutant removal can be explained by the complex relationships between influential soil and water characteristics. Because of its complexity, conventional mathematical or statistical parameters find it difficult to simulate accurately the relationship between system properties and removal efficiency. Thus, machine learning may offer an alternative to conventional mathematical techniques for modeling pollutant removal with MAR. Many applications of environmental engineering have effectively utilized data-driven modeling. Advanced optimization tools were applied to improve process efficiency as well as minimize computation cost and material requirements (Eren et al., 2019; Bhagat et al. 2020; Yaqub et al. 2021). Despite numerous publications describing the application of ML models in water treatment systems, studies on the creation of models that forecast MAR’s success in removing pollutants for wastewater reclamation and reuse are scant (Yaqub and Lee 2020). Yaqub and Lee (2020) used MLPNN as a black box study with no equations derivable; GEP allows for the creation of an ET which is utilized to create a function that represents the correlation between the inputs and outputs. As a result, GEP is superior in predicting pollutant removal via MAR and can reduce the experimental workload. Gorski et al. (2022) developed a boosted regression tree model to forecast nitrate removal during infiltration, pinpointing urban-agricultural interfaces as ideal managed aquifer recharge locations. Liu et al. (2020) integrated logistic regression with ML to forecast groundwater penetration into sewage systems, with an accuracy of 82%. Kumar and Sihag (2019) evaluated empirical and ML models for predicting soil infiltration rates, concluding that random forest regression was the most effective. These studies illustrate the capability of ML in enhancing MAR site selection and forecasting infiltration dynamics. Recent research has investigated ML methodologies for comprehending and forecasting groundwater dynamics. Random forest algorithms have demonstrated significant precision in calculating groundwater withdrawals through the use of multitemporal satellite data and water balance components (Majumdar et al. 2020). Multiple ML algorithms, such as SVM, multivariate adaptive regression splines, and RF, have been utilized to delineate groundwater recharge potential zones, with RF exhibiting greater efficacy (Pourghasemi et al. 2020). Statistical learning techniques, including RF and XGM, have been employed to create predictors for aquifer recharge, including data uncertainty and noise (Martin & Yang 2023). Traditional machine learning classification methods, such as classification and regression tree (CART), have been utilized to quantify groundwater recharge threshold conditions throughout Australia, offering insights into recharge mechanisms and spatial variability (Hu et al. 2023). Exhibit the capabilities of ML in enhancing MAR site selection and forecasting infiltration dynamics. Sahoo (2022) established an ensemble modeling framework that integrates spectral analysis and machine learning to forecast groundwater level fluctuations in agricultural areas, identifying irrigation demand as the predominant cause. Jain et al. (2023) employed ML methodologies to examine the correlation among alterations in surface water, land surface temperature, and rainfall intensity in India. Pourghasemi et al. (2020) evaluated three ML algorithms (SVM, MARS, and RF) for delineating groundwater recharge potential zones, with the RF model exhibiting the greatest accuracy. These works illustrate the efficacy of ML methodologies in comprehending and forecasting surface water and groundwater dynamics, providing essential instruments for water resource management. According to predictive ML models, the potential of “surprise” is considerable. Under some circumstances, even the greatest algorithms, mathematical frameworks, and analytical instruments can result in total failure. The corresponding abbreviations of the manuscript are reported in the Nomenclature. Table 4 reports the ML applications for implementation of MAR for specific conditions with their limitations.

**Table 4:** ML applications for implementation of MAR for specific conditions with their limitations

Specific issue	ML	Data used	Model metrics & performance	Limitation	Reference
Groundwater recharge potential maps	SVM, MLA, LASSO, & RF	Elevation, aspect, slope angle, TWI (topographic wetness index), fault density, MRVBF (multiresolution index of valley bottom flatness), rainfall, lithology, land use, drainage density, distance from rivers, distance from faults, annual, ETP (evapotranspiration), minimum temperature, maximum temperature, and rainfall 24 h	Based on the validation, the RF algorithm performed better (AUC = 0.987) than the SVM (AUC = 0.963) and the MARS algorithm (AUC = 0.962). Furthermore, based on the ROC curve threshold, the accuracy of these MLAs is included in the excellent class	Identifying suitable places for groundwater replenishment, equal to groundwater abstraction, can help sustain groundwater resources globally. In order, evaluating the most robust and appropriate MLA is crucial for creating an exact GRPM	Pourghasemi et al., (2020)
Water and groundwater quality indices (WQI and GWQI), water pollution index (WPI)	non-parametric kernel Gaussian learning (GPR), ANFIS), DT	pH, oxidation–reduction potential (ORP), electrical conductivity (EC), total dissolved solids (TDS), turbidity, dissolved oxygen (DO), temperature, and atmospheric pressure	The findings demonstrated that GPR displayed remarkable accuracy during the testing phase, with an RMSE of 0.0169 for GWQI. Likewise, for WPI, the ANFIS demonstrated significant predictive accuracy throughout testing, with an RMSE of 0.0401	The ANFIS models typically surpass DT models, however, they are often comparable to or occasionally outperformed by GPR models, especially during the testing phase, highlighting GPR’s robustness in generalization	Jibrin et al. 2024
Water level prediction	LR, SVM, GPR, and NN	Scenarios based on the two monthly historical variables (groundwater and water level) for 100 monthly data sets from 2012 to 2019	Matern 5/2 of Gaussian Processes Regression Models is the most accurate water level predictor, performing well in all scenarios except SC3 with a rapid training period	The study’s limitations are comparing the performance and resilience of parametric and nonparametric approaches across various circumstances and examining the uncertainty of the detected modes	Sapitang et al. 2021
Climate change scenarios	Linear Regression, Ridge Regression, LSTM, MLP, Polynomial Regression, RF Regressor, DR Regressor, GBR, XGBoost, and Light Gradient Boosting Model (LGBM)	Precipitation, land use land cover (LULC), soil type, land slope, temperature, potential evapotranspiration, and aridity index (*ArIn*) from 1986 to 2019	The application of statistical parameters to all created models indicated that the XGBoost model had superior prediction drives and generalization to unknown data, characterized by low MSE, high adjusted *R*^2^ scores, low MAE, and high NSE	Scalability, computational efficiency, parallel processing, and autonomous feature selection make XGBoost suited for huge datasets and uncertain data settings, capturing nonlinear aspects is challenging. An extensive uncertainty analysis was done to understand model prediction uncertainty	Banerjee et al., 2024
Water balance	Classification and Regression Tree (CART), Random Forest (RF), and Logistical Regression (LR),	Rainfall, evaporation, soil moisture, runoff, and vegetation	Utilising the Australian continent as a case study, CART is the most expedient technique, yielding an average classification accuracy of 76%, which is comparable to RF at 77–78% and LR at 79–80%. Concurrently, CART is the sole methodology that ranks the significance of conditions while establishing a distinct threshold for each condition	The trustworthiness of the disclosed groundwater recharging mechanism is the primary problem, as this work seeks to replicate model-simulated recharge instead of actual recharge. Inaccurate predictions provide a further concern regarding the dependability of the disclosed mechanism	Hu et al. 2023

#### Case study on implementing ML in MAR

**Fig. 6:**
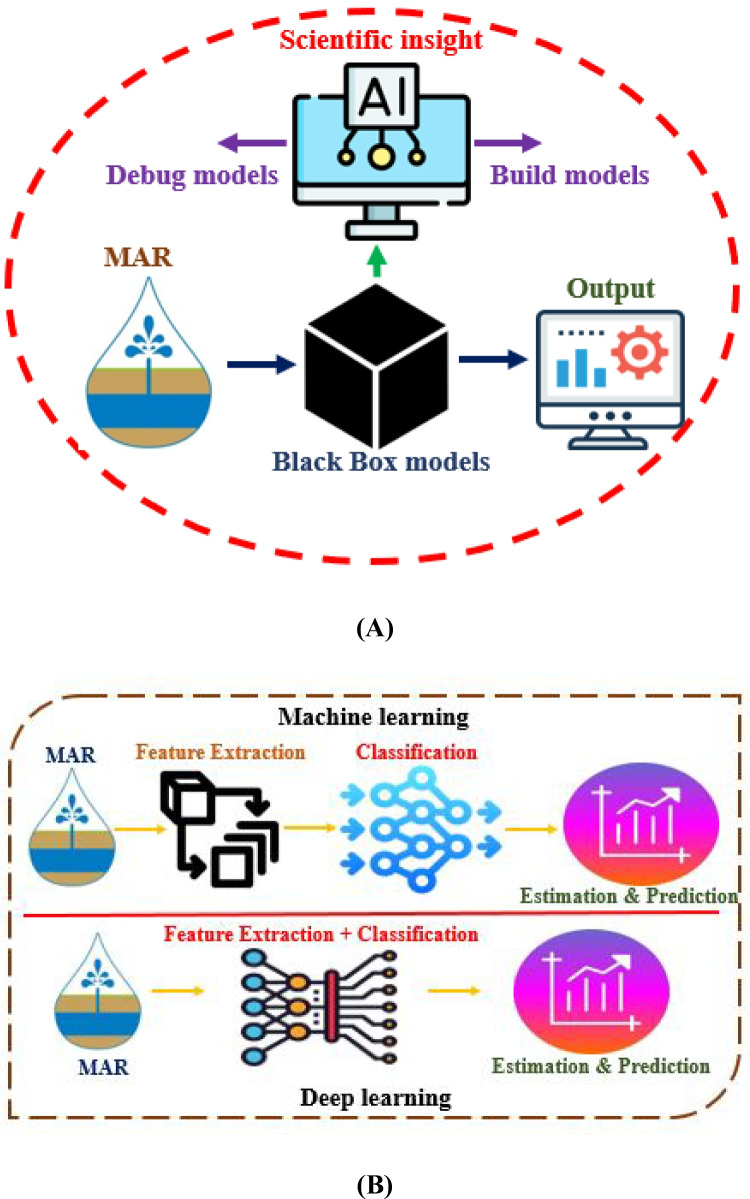
**A** Interpreting machine learning models, and **B** machine and deep learning models for MAR

The artificial neural network (ANN) model was created for groundwater modeling by utilizing nine input data layers, encompassing lithology, land use, proximity to major rivers, precipitation, water quality, soil transmissivity, and slope maps. This study demonstrates that the prediction accuracy attained using the fuzzy analytic hierarchy process (FAHP) method is significantly higher when compared to ANN methods. In contrast to the FAHP model, the ML model demonstrated superior accuracy and more cost-effective computational expenses (Mojtaba Zaresefat et al. 2023). This study provides practical guidance and innovative ideas for professionals and policy planners in the search for suitable MAR regions. Various ML techniques, including SVM and decision tree-RF, can be compared with the proposed approach. Future research should evaluate the suggested techniques against existing practices to identify new locations for sustainable MAR, enhancing efficiency while minimizing time and resource consumption. California serves as a prospective case study for applying ML to aquifer recharge management (Escriva-Bou et al. 2020). Prolonged severe drought in California has led to declining groundwater levels and increased water shortages. The state has initiated several MAR activities to enhance water storage in underground aquifers. ML can analyze data sources such as geological, hydrological, and climatic data to determine optimal MAR sites. By utilizing historical rainfall data, ML algorithms can predict regions likely to experience heavy precipitation in the future, aiding in the identification of successful MAR areas (Zhang et al. 2020). ML can also assist in the optimal management of selected MAR sites, predicting water availability throughout the year and enabling precise recharging schedules. ML algorithms can analyze data to identify potential MAR issues based on trends and patterns, and sensor installations can monitor groundwater levels. Another advantage of ML is its ability to monitor the long-term performance of MAR sites. However, despite their significant potential for prediction and control in MAR, few research articles specifically emphasize the use of ML and DL (interpretable ML, LSTM, BiLSTM, etc.) algorithms. Figure 6(A) illustrates the interpretation of ML models, while Fig. 6(B) showcases the models for ML and DL in the context of MAR.

### Future recommendations, challenges, limitations, and improvements

#### Future recommendations

It has been demonstrated that MAR techniques play a crucial role in alleviating or preventing water crises by intentionally injecting subsurface water into aquifers for future release. Consequently, thorough research is imperative to address all facets of MAR. This paper reviews the role and factors affecting the effectiveness of MAR and presents an overview of current research activities while forecasting future advancements utilizing ML and DL. Future notable recommendations for research related to MAR and utilization of data analytical tools are listed below:

- A systematic exploration of clogging mechanisms involving physical, chemical, and biological factors, including preferential flow concepts, is essential. Developing more effective and user-friendly methods for assessing, predicting, and preventing clogging is an ongoing necessity, with potential future research directions exploring microbial solutions to clogging issues.
- Enhanced understanding of the theory behind MAR seepage calculations is crucial for ensuring GWM security. Multidisciplinary research involving hydrogeology, ecology, and microbiology integration is essential for addressing the current demand for a comprehensive understanding of MAR purification mechanisms.
- The integration of big data analytics in MAR is anticipated to support future advancements and facilitate information sharing. Despite the analysis of ML and DL fault detection and diagnosis algorithms in process engineering, MAR applications in this domain are limited. More detailed explorations are required on DL models and interpreting ML models, particularly in the context of digital twins and their application in MAR. Very few research focused on the applications of ANN and genetic algorithms in the mapping of MAR as well as GW level (Mojtaba Zaresefat et al. 2023; Salehi Shafa et al. 2023).
- Researchers should focus on developing DL models and interpreting ML models for a better understanding of MAR processes. Techniques such as partial dependence plots (PDPs), Shapley additive explanations (SHAPs), feature importance factors (FI), and variance inflation factors (VIF) are valuable for gaining insights into model predictions and enhancing human decision-making (Molnar 2020; Park et al. 2022). Future studies should consider these methods to improve the performance and control of MAR. Notably, boosted trees, including XGBoost (XGB) and distributed gradient-boosted decision tree (GBDT), provided a more accurate description of WQ compared to generative additive models (GAM), as reported by Schäfer et al. (2022). These approaches demonstrate the capability to streamline input variables, which proves beneficial for computationally intensive algorithms like multi-output Gaussian process regression (Srungavarapu et al. 2023), among others.

#### Challenges of ML in applying MAR

Effective groundwater management in MAR can benefit from the application of ML. However, the implementation of ML is speculated to pose several challenges that must be overcome for optimal effectiveness. The key challenges require attention as elaborated below:

- ML algorithms demand large amounts of high-quality data for accurate training and prediction. However, GWM often faces challenges in obtaining extensive and reliable data, especially in developing or remote areas. Issues such as inaccuracies, inconsistencies, or outdated data further complicate the task of ensuring data quality, making this a significant challenge.
- After data collection, employing data science techniques for cleaning, error removal, and handling missing data is crucial. Additionally, the selection of suitable ML algorithms can be challenging, given the multitude of options available. Identifying the most appropriate algorithms for groundwater-related data requires further study.
- Integrating ML and DL with diverse data sources can be challenging. ML systems need to handle various data formats, resolve inconsistencies, and combine information from different sources for accurate insights into groundwater management. Transparency and explainability are essential for informed decision-making and building trust among stakeholders. This challenge can be addressed by using interpretable ML-based models or by combining ML with traditional hydrological models.
- Implementing ML in MAR may demand substantial computational resources, technical expertise, and financial investment. Affordability issues related to ML technologies may hinder adoption, particularly in regions with limited resources. Addressing these resource constraints and providing support for capacity building in GWM is crucial for widespread ML adoption. Establishing clear standards and norms for the responsible and ethical use of ML technology is essential. This includes considerations related to data governance, transparency, and fairness, ensuring that ML applications align with ethical principles in groundwater management.

#### Limitations of MAR in comparison to other fields by using AI applications

Within the realm of MAR management, it is imperative to not only predict outcomes but also comprehend the fundamental mechanisms that accelerate those results. A lack of explainability in an AI model’s prediction process can generate skepticism and hesitancy in accepting the model’s recommendations. This issue is especially troublesome in the context of MAR, as the decisions made might have enduring effects on both the availability and WQ (Ahmad et al., 2023). AI could improve priority-based scheduling in MAR, but it has many limitations. These include complexity, interpretability, interaction with existing systems, generalization and transferability constraints, computational demand, and regulatory difficulties. AI can only be integrated into MAR to improve groundwater management efficiency, equity, and sustainability by addressing these hurdles (Cai et al. 2024). AI could optimize injection rates and recharge column quality in MAR systems. AI can optimize column design, extend operational life, and dynamically alter injection rates to maximize recharge efficiency and prevent groundwater mounding using data-driven insights. AI must be applied properly in MAR initiatives by managing data dependencies, complexity, and integration problems (Usui et al. 2024). AI can assist in securing groundwater resources for future generations by solving these limits and improving MAR sustainability and efficacy.

#### Potential improvements of ML in the application of MAR

- Pay attention to the models’ capacity to learn from small datasets. Metrics that assess a model’s performance with limited data include accuracy and F1 score for few-shot tasks. Determines the degree to which a pre-trained model may be easily adjusted to new tasks or domains. This metric’s improvements enable the more effective application of current models in novel settings. Analyze a model’s performance when it has fewer labeled data points. By enhancing these criteria, it will be possible for ML models to operate well without requiring a lot of labeling work.
- Assesses the precision of forecasted probability. Improvements in this score can enhance models in probabilistic prediction tasks (e.g., MAR site meteorological forecasting). Evaluate the correspondence between expected probability and actual results. Enhanced calibration metrics guarantee that confidence estimations (e.g., probabilities in classification) are more dependable and practical. Metrics such as expected uncertainty quantify the extent of uncertainty in forecasts.
- Metric guarantees the equivalence of the true positive rate (TPR) and false positive rate (FPR) among various demographic groupings. Enhancing these measures ensures that models do not unjustly advantage or disadvantage any group. Enhancing this metric guarantees that machine learning models do not perpetuate systemic biases.
- The meticulous identification and monitoring of pertinent performance metrics can substantially enhance the incorporation of ML in MAR. Metrics include prediction accuracy (MAE, RMSE), optimization performance (objective function value), water quality (precision, recall), and operational efficiency (recharge efficiency, energy consumption per unit of water) that facilitate the evaluation of the efficacy of machine learning in improving managed aquifer recharge systems. These criteria guarantee that ML models may be optimized to enhance water recharge, mitigate hazards, maximize energy efficiency, and preserve long-term groundwater sustainability.

## Conclusion

In summary, the application of ML models has proven highly effective in capturing the complex hydrogeological relationships between input variables, groundwater levels, and concentrations. These models excel in predicting groundwater levels and contamination, even with incomplete or inaccurate data, due to advances in clustering and ML techniques. They have led to the development of robust decision support tools for predicting groundwater levels and contamination across varied water resource management scenarios. It is recommended to perform clustering of wells, stations, and sampling points before applying ML techniques, particularly in heterogeneous systems. This review highlights the following implications for MAR:

- ML enables the processing of extensive sensor and satellite data for real-time monitoring of groundwater resources, detecting issues like declining water levels or contamination that might be missed by human observation.
- Advanced ML models use historical and environmental data to predict groundwater behavior, identify areas prone to drought, and assess the impacts of climate change.
- ML applications allow for the optimization of MAR strategies by integrating multiple variables such as water demand, aquifer characteristics, and regulatory requirements.
- ML systems can detect early warning signs such as contamination, salinization, and over-pumping, facilitating proactive measures to mitigate long-term damage to groundwater resources.
- ML improves decision-making by integrating data from diverse sources and scenarios, optimizing maintenance schedules, detecting leaks, and identifying vulnerable infrastructure in water distribution systems.

The successful implementation of ML requires addressing several challenges. The acquisition and quality of groundwater-related data, particularly in less-developed or remote areas, present a significant hurdle. Additionally, the complexities of data cleaning and algorithm selection underscore the need for thorough studies to determine the most suitable ML approaches. Expertise and judgment remain essential for validating and interpreting ML outputs, ultimately informing sound decision-making processes. The application of ML in MAR has a great deal of potential, but ethical considerations, transparency, and accountability must be taken into account. For ML outputs to be validated and interpreted, and for final decisions to be made, experts and judgment are essential.

## Supplementary information

Below is the link to the electronic supplementary material.

ESM 1 (DOCX 29.4 KB)

## Data Availability

The information and conclusions presented in this review are based on previously published studies, and references to these sources are provided in the bibliography. The authors confirm that the data supporting the findings of this study are available within the article.

## Nomenclatures

AAD: Absolute average deviation
ASR: Aquifer storage and recovery
AM: Aquifer matrix
ABRF: AdaBoost random forest
AC: Accuracy
ACO: Ant colony optimization
AIG: Algorithm of Innovative Gunner
ANFIS: Adaptive neuro-fuzzy inference system
BF: Hybrid beamforming
BMP: Best management practices
BRT: Boosted regression tree
BiLSTM: Bidirectional long short-term memory
BN: Bayesian networks
BRF: Bagging random forest
BWO: Black Widow optimization
CC: Coefficient of correction
Cl: Chloride (mg/L)
DT: Decision tree
DI: Diclofenac (ng/L)
DNN: Deep neural network
DOC: Dissolved organic carbon (mg/L)
EC: Electrical conductivity (μS/cm)
ELMNN: Extreme learning machine neural network
EGB: Extreme gradient boosting
FA: Factor analysis
Fe: Ferrous (mg/L)
FL: Fuzzy logic
FFNN: Feed-forward neural network
FMF: Fuzzy membership framework
GEP: Gene expression programming
GA: Genetic algorithm
GFI: Groundwater footprint index
GPR: Gaussian process regression
GRNN: Generalized regression neural network
GMDHM: Group method of data handling model
HHO: Harris hawk’s optimization
IP: Iopromide (ng/L)
ICA: Independent component analysis
LSSVR: Least-squares support vector regression
LBRF: LogitBoost random forest
MLPNN: Multilayer perceptron neural network
Mn: Manganese (mg/L)
MF: Mean fitness
MLR: Multiple linear regression
MFT: Multi-frameworks technique
MAPE: Mean absolute percentage error
MMA: Multi-models approach
MLP: Multi-layer perceptron neural network
MSE: Mean square error
MAE: Mean absolute error
RMSE: Root mean square error
NN: Neural network
NARXNN: Nonlinear autoregressive exogenous
NO_2_: Nitrogen dioxide (mg/L)
NO_3_: Nitrate (mg/L)
NSGA-II: Multi-objective genetic algorithm
PPV: Positive predictive value
PS: Particle swam
PCA: Principal component analysis
PP: Pharmaceutical products
Pr: Propranolol (ng/L)
RF: Random forest
RBFNN: Radial basis function neural network
SATS: Soil aquifer treatment systems
SSE: Sum of square-error
SDF: Standard DRASTIC-Fr framework
SC: Screened casing
SO_4_^2−^: Sulfate (mg/L)
SES: Single exponential smoothing
SVM: Support vector machine
SLA: Superposition-based learning algorithm
SP: Specificity
SE: Sensitivity
SF: Surface water
R2: Coefficient of determination
RVM: Relevant Vector Machine
Tr: Trimethoprim
WO: Whale optimization
XGB: Xtreme gradient boost
VZIF: Vadose zone infiltration
