# Supplementary material for: Machine learning-based monitoring and design of managed aquifer rechargers for sustainable groundwater management: scope and challenges
Source: Environ Sci Pollut Res Int. 2024 Nov 25;32(59):31572–605. doi: 10.1007/s11356-024-35529-3 (PMC12819460; doi:10.1007/s11356-024-35529-3)
Supplement: Supplementary file 1 — (DOCX 29.4 KB) [file 11356_2024_35529_MOESM1_ESM.docx]

**Machine Learning-Based Monitoring and Design of Managed Aquifer Rechargers for Sustainable Groundwater Management: Scope and Challenges**

Abdul Gaffar Sheik^a^, Arvind Kumar^a^, Anandan Govindan Sharanya^b^, Seshagiri Rao Amabati^c^, Faizal Bux^a^, Sheena Kumari ^a*^

^a^Institute for Water and Wastewater Technology, Durban University of Technology, Durban-4001, South Africa.

^b^Department of Civil Engineering, Indian Institute of Technology Bombay, Mumbai 400076, India.

^c^Department of Chemical Engineering, Indian Institute of Petroleum and Energy, Visakhapatnam – 530 003, Andhra Pradesh, India

***Corresponding Authors Email:** [Sheenak1@dut.ac.za](mailto:Sheenak1@dut.ac.za)

| **Table S1** ML performance metrics for GWM in MAR | | | | |
| --- | --- | --- | --- | --- |
| **Indices** | **Referred Eq** | **Target Value** | **Citation** |  |
| $\mathrm{MSE}$ | $\frac{\sum\left( z_{i}-y_{i} \right)^{2}}{n}$ | As long as the model does not have any errors and makes better predictions, the MSE is equal to zero. | (Che Nordin et al. 2021; Hanoon et al. 2021; Gholami et al. 2022; Siabi et al. 2022) |  |
| RMSE | $\sqrt{\frac{\sum_{i=1}^{n} \left( z_{i}-y_{i} \right)^{2}}{n}}$ | Models with zero errors and better predictions have a zero MSE.A good fit is therefore indicated by values near 0. |  |  |
| SSE | $\sum_{i=1}^{n} \left( x_{i}-x_{mean} \right)$ | The prediction is more precise when the SSE is lower. It indicates that the values close to 0 are well-fitting. | (Singha et al. 2021); (Stackelberg et al. 2021) |  |
| R2 | $1-\frac{\sum_{i=1}^{n} \left( z_{i}-y_{i} \right)}{\sum_{i=1}^{n} \left( z_{i}-y_{mean} \right)}$ | A value that is close to 1 has a better prediction. It ranges between 0 and 1. | (Kadam et al. 2019; Dehghani and Torabi Poudeh 2022) |  |
| AAD | $\frac{\sum_{i=1}^{n} \left( x_{i}-x_{mean} \right)}{n}$ | Greater values indicate a greater variation of the data points from the mean. So, when value is little, the best fit will be attained. | (Msaddek et al. 2022) |  |
| Adjusted R2 | $\frac{\left( 1-R^{2} \right)x\left( n-1 \right)}{\left( n-k-1 \right)}$ | Better prediction is made when the value is close to 1. It ranges between 0 and 1. | (Dehghani and Torabi Poudeh 2022) |  |
| NRMSE | $\frac{\sum_{i=1}^{n} \left( z_{i}-y_{i} \right)^{2}}{\sum_{i=1}^{n} y^{2}}$ | A model's ability to "fit" a dataset improves with decreasing RMSE | (Singha et al. 2021) |  |
| MAE | $\frac{\sum_{i=1}^{n} \left( z_{i}-y_{i} \right)}{n}$ | Same as NRMSE | (Al-Adhaileh et al. 2022) |  |

n - total number (t_n_) of data points, y_i_- actual data, z_i_ is forecasted value, k -tn of parameters, R defines maximal pixel value of the image, w is the weight of each performance index, a, r, and f represent accuracy, recall, and F-measure, true positive (TP), negative (TN), and false negative (FN).
